# Determination of Therapeutic and Safety Effects of *Zygophyllum coccineum* Extract in Induced Inflammation in Rats

**DOI:** 10.1155/2022/7513155

**Published:** 2022-07-18

**Authors:** Mohammed Yosri, Mahmoud M. Elaasser, Marwa M. Abdel-Aziz, Marwa M. Hassan, Abdulmohsen Hussen Alqhtani, Naif Al-Gabri, Ahmed B. A. Ali, A. Pokoo-Aikins, Basma H. Amin

**Affiliations:** ^1^The Regional Center for Mycology and Biotechnology, Al Azhar University, Nasr City, Cairo, Egypt; ^2^Anatomy and Embryology Department, Faculty of Medicine, Helwan University, Egypt; ^3^Animal Production Department, Food and Agriculture Sciences College, King Saud University, Riyadh, Saudi Arabia; ^4^Department of Pathology, Faculty of Veterinary Medicine, Thamar University, Yemen; ^5^Laboratory of Pathology, Salam Veterinary Group, Buraydah, Al Qassim 51911, Saudi Arabia; ^6^Department of Animal and Veterinary Science, Clemson University, SC, USA; ^7^US National Poultry Research Center, Toxicology & Mycotoxin Research Unit, USDA, ARS, Athens, GA 30605, USA

## Abstract

**Background:**

*Z. coccineum* is a facultative plant with many medicinal applications. This study examined the anti-inflammatory activity of *Zygophyllum coccineum* (*Z. coccineum*) in an arthritis animal model.

**Materials and Methods:**

Seventy-Six Wistar Albino rats of either sex randomly divided into six groups (12/each). The inflammation model was done using Complete Freund's Adjuvant in albino rats. The anti-inflammatory activities of the extract were estimated at different dose levels (15.6, 31, and 60 mg/kg) as well as upon using methotrexate (MTX) as a standard drug (0.3 mg/kg). Paw volume and arthritis index scores have been tested in all examined animals' treatments. Histological examination of joints was also performed. Flow cytometric studies were done to isolated osteoclasts. Cytokines assay as well as biochemical testing was done in the examined samples. *Results. In vitro* studies reported an IC_50_ of 15.6 *μ*g/ml for *Z. coccineum* extract in lipoxygenase inhibition assay (L.O.X.). Moreover, it could be noticed that isorhamnetin-3-O-glucoside, tribuloside, and 7-acetoxy-4-methyl coumarin were the most common compounds in *Z. coccineum* extract separated using L.C.–ESI-TOF–M.S. (liquid chromatography-electrospray ionization ion-trap time-of-flight mass spectrometry). Microscopic examinations of synovial tissue and hind limb muscles revealed the effect of different doses of *Z. coccineum* extract on restoring chondrocytes and muscles structures. Osteoclast size and apoptotic rate examinations revealed the protective effect of *Z. coccineum* extract on osteoclast. The results upon induction of animals and upon treatment using of MTX significantly increased apoptotic rate of osteoclast compared to control, while using of 15.6 *μ*g/ml. for *Z. coccineum* extract lead to recover regular apoptotic rate demonstrating the protective effect of the extract. *Z. coccineum* extract regulated the secretion of proinflammatory and anti-inflammatory cytokines. Biochemical tests indicated the safety of *Z. coccineum* extract on kidney and liver functions. *Conclusion. Z. coccineum* extract has efficient and safe anti-inflammatory potential in an induced rat model.

## 1. Introduction

Rheumatoid arthritis (R.A.) is a form of arthritis that leads to pain, swelling, and distortion of joints functions. It is a long-lived disorder developed by combined genetic, epigenetic, and environmental circumstances. Many immune and nonimmune cells, as well as inflammatory factors, have essential functions in the process of inflammation and joint destruction [1]. The synovium considers as potential tissue in R.A. [2], and the formation of a pannus leads to the destruction of cartilage and bone. Chondrocytes are affected by many autoantibodies and secreted extracellular components [3]. Osteoclasts are multinucleated cells, and they act as bone modulator cells. They are generated in the bone marrow through the stimulatory effects of macrophage colony-stimulating factor (M-CSF) and receptor activator of nuclear factor *κ*B ligand (R.A.N.K.L.) [4–6]. Cytokines are pivotal in both humoral and adaptive responses in developing rheumatoid arthritis [7]. The equilibrium in pro and anti-inflammatory cytokines using either natural or synthetic factors is innovative in overcoming rheumatoid arthritis pathology [8].

The available nonsteroidal anti-inflammatory drugs to treat rheumatoid arthritis have many drawbacks, including abdominal discomfort in many internal body organs affecting their functions. Alternative therapy is a modern path that has been used to combat many human diseases. It has been reported that 50-80% of rheumatoid arthritis patients use conventional herbal therapies [9–11]. Different natural extracts have been used with various beneficial activities including regulating liver damage [12–15], various types of cancer cells [16, 17], diabetes [18], and COIVID-19 [19], through regulating various signaling pathways [20–23].

*Z. coccineum* is a succulent shrub from the family Zygophyllaceae. It can grow in different kinds of soil and can tolerate high salt levels inland. It is distributed in many African, Asian, and Mediterranean areas as well as Australia [24–26]. The aerial parts contained bioactive molecules with promising antifungal and insecticidal actions [27, 28]. It has been used in the removal of high content of heavy metals, including copper and lead, in water [29]. *Z. coccineum* extract has been tested against induced diabetic rats, showing promising activity to control high blood glucose with minimal burden in body organs, including liver and kidney [30]. *Z. coccineum* aqueous extract was reported to control elevated blood pressure and heart rate in induced rats [31]. In this study, the anti-inflammatory properties of *Z. coccineum* extract have been investigated *in vitro* and in induced rats, illustrating its therapeutic effects with a noticeable safety profile.

## 2. Materials and Methods

### 2.1. Preparation of Extract

*Z. coccineum* was collected in 2020 from the deltaic coast of Egypt and identified by the institutional plant taxonomist (Prof. Abbas Elgamry) at the Botany and Microbiology Department, Faculty of Science, Al Azhar University according to (Voucher No. ID: 0044). 500 gm whole plant was air-dried, crushed with blinder extracted from aq.-ethanol 70%, dried, and were stored at -20°C for further analysis [32].

### 2.2. Determination of Phytochemicals

Separation was achieved on a 5 *μ*m C18 column (50 × 2.0 mm internal diameter; Bohus, Sweden; 45°C) using Agilent L.C.–ESI-TOF–M.S. (Agilent Technologies, Palo Alto, CA, U.S.A.) System. The LC mobile phases consisted of (A) 5 mM NH_4_HCO_2_ in 1% CH_3_OH (pH 3.0) and (B) 5 mM NH_4_HCO_2_ in 1% CH_3_OH (pH 8.0). Gradient elution was done at 0.2 ml/min and 0–20 min, 10% B; 21–35 min, 90% B; 35.01–60 min, 10% B; and then 90% B to the end of the run. The sample was injected for LC-MS analysis in both positive and negative modes. The LC-MS analysis was also performed before the sample injection for blank and quality control samples for confidence in the experiment. Infusion experiments were carried out to optimize the negative ion ESI-MS/MS parameters for maximal generation of deprotonated molecules and effective generation of characteristic fragment ions for all analyses. The MS-DIAL V. 3.70 open-source software was used for the identification and calculation of the relative percentage of compounds [32, 33]. MS-DIAL 3.70 software was used for small molecule analysis of the separated sample. According to the acquisition mode, ReSpect positive (2737 records) or ReSpect negative (1573 records) databases were used as reference databases [34].

### 2.3. In Vitro Lipoxygenase (L.O.X.) Inhibition Assay

*Z. coccineum* extract and the reference compound (Ibuprofen) were tested in order to investigate the anti-inflammatory response by inhibiting the L.O.X. enzyme (lipoxygenase enzyme) from Glycine max (type I-B) according to Granica et al. [35] with slight modifications. Briefly, in 96-well plates, 100 *μ*l of soybean L.O.X. solution (1000 U/ml in borate buffer solution, pH 9) and 200 *μ*l of borate buffer were mixed together with varying concentrations of the sample to a final concentration range of 0.98-125 *μ*g/ml at 25°C for 15 min. Samples were preincubated with 100 *μ*l of linoleic acid (substrate) to start the reaction. The inhibitory activity was determined by monitoring the absorbance's increase at 234 nm using a microplate reader (BioTek, U.S.A.).

### 2.4. Animals and Treatments

Male Wister albino rats (10 weeks old) weighing 130–150 g were purchased from the animal unit of Faculty of Science, Al-Azhar University, left for acclimation for ten days, and split into six groups (twelve rats each). The first group was observed as negative control (N.C.) infused subcutaneously with saline and 10% Tween-80, twice per week for two weeks. The other groups were interjected subcutaneously twice a week for two weeks at the base of the tail with 100 *μ*L C.F.A. (Complete Freund's Adjuvant) (Sigma-Aldrich, U.S.A.) to convene arthritis model [36]. The second group presents the positive control (P.C.) that was kept untreated. Three estimated doses (15.6, 31, and 60 mg/kg) of *Z. coccineum* extract were administrated subcutaneously in the third, fourth, and fifth groups, respectively. Lastly, antirheumatic standard drug MTX (Methotrexate) (Orion Pharma, Espoo, Finland) was injected. Signs of arthritis were raised after 14 days, and then *Z. coccineum* extract was used three times per week for 2 weeks at a dose of 15.6, 31, and 60 mg/kg extract B.W. MTX was used subcutaneously twice per week at a dose of 0.3 mg/Kg B.W. [37] as shown in supplement (1). The animal studies were approved by the ethical committee in the Regional Center for Mycology and Biotechnology (No. RCMB26062020).

### 2.5. Assessing Swelling Scoring

Severity scores and paw volume were assessed daily to evaluate inflammation. The severity score test is graded on a scale of 0–5, where a total score of 5 points indicates severe inflammation deficits and a score of 0 indicates normal performance; 4 points indicates severe injury, 3 indicates mean to moderate injury, and 1-2 indicate mild injury. Evaluation was performed after induction by technicians who were blinded to the experiments [38]. Paw volumes have been measured using a digital plethysmometer (B.V.K., India) [39].

### 2.6. Histopathology Studies

Animals were euthanized by cervical dislocation. Tibiofemoral joint and hind limb muscles were harvested, for tibiofemoral joint samples only that were decalcified using 5% nitric acid for 10 days. There are both types of samples (joints and muscles). Sections of samples were cut at 5 *μ*m on a rotary microtome, processed, mounted on slides, and stained (Leica Autostainer XL) with hematoxylin and eosin, to assess tissue morphology and imaged at ×20 (Zeiss microscope and imaging system, Carl Zeiss Inc., Germany) [40].

### 2.7. Transmission Electron Microscopy

To test ultrastructure changes of decalcified tibiofemoral joint and hind limb muscles, transmission electron microscopy was used. Samples chemically fixed in an aqueous solution of glutaraldehyde (2.5%) for two days at 4°C, followed by postfixation with a 1% osmium tetroxide solution for four hours at 4°C followed by sectioned using ultra-microtome (Leica, Germany) and analyzed using 1010 T.E.M. microscopy (J.E.O.L., Japan) in the Regional Center for Mycology and Biotechnology [41].

### 2.8. Cell Culture Studies

Bone marrow-derived monocytes/macrophages were detached from the tibias of male rats by rub the bone-marrow hole with D.M.E.M. enriched by 10% fetal bovine serum, 5% l-glutamine, 100 U ml−1 penicillin, and 100 *μ*g ml−1 streptomycin. The cells were bred for six h to split nonadherent and adherent cells into six well tissue culture plates. Nonadherent cells were cultured in 6-well tissue culture plates at 2 × 105 cells/well in the presence of 10 ng/ml rh M-CSF (Invitrogen, U.S.A.) for 3 days to collect macrophage-like osteoclast precursor cells. After 3 days, the nonadherent cells were collected, and preosteoclasts were cultivated in the existence of 10 ng/ml M-CSF, 50 ng/ml R.A.N.K.L., and diverse levels of sodium butyrate for 4 days to form osteoclasts. On day 2, the medium was replaced with fresh medium consisting of M-CSF, R.A.N.K.L., and sodium butyrate. Photos were captured using inverted microscopy Zeiss microscope and imaging system (Carl Zeiss Inc., Germany) [42].

### 2.9. Flow Cytometry Analysis

Cultured osteoclasts in 6-well plate were used for this test. Osteoclasts were split using trypsin in 0.25% pancreatin and washed using phosphate-buffered saline. The death rate was assessed by an Annexin V-FITC and propidium iodide (P.I.) staining kit (B.D. Bioscience, U.S.A.), and cells were suspended using buffer which has Annexin V-FITC and/or P.I. stock solution and kept away from light at room temperature for 15 min. The cells were examined by flow cytometry (B.D. Bioscience, U.S.A.) [43, 44].

### 2.10. ELISA Testing

Blood samples were collected from rat's eyes in heparinized Eppendorf tubes before sacrificing animals and centrifuged at 5000 rpm for 15 min at 5°C. Then serum was collected and kept at -80°C till analysis. Serum levels of IFN-*γ*, IL-1*β*, IgG1a, IgG2a, IL4, IL-6, and IL-17 were detected by kits (Abcam, U.S.A.) using the manufacturer's explained steps.

### 2.11. Biochemical Tests

The levels of A.L.T., creatinine, and C-reactive protein in serum were determined by the standard protocol using the biochemical diagnostic kits (Diamond, UK) [45].

### 2.12. Statistical Analysis

Calculations of statistical variance were done by GraphPad Prism 5 software (San Diego, CA). Either two groups were represented in the test, an unpaired two-sided Student's *t*-test was done; for relations of more than two groups, one-way analysis (ANOVA) was used followed by Turkey's post-hoc test to detect significance between groups where *P* < 0.05 is expressed significant.

## 3. Results

### 3.1. LC–ESI-TOF–M.S. Analysis

**Figure 1:**
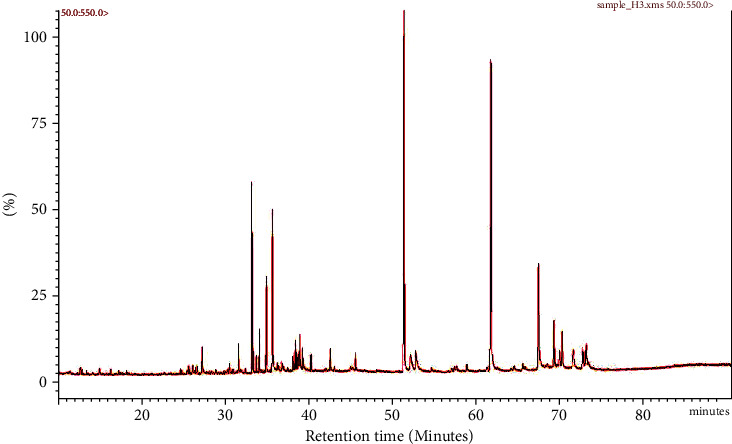
Total ion chromatograph (TIC) of separated compounds in the aqueous-ethanolic extract of *Z. coccineum* detected by L.C.–ESI-TOF–M.S. Each separated peak represents a chemical constituent at specified retention time.

The aq.-ethanolic extract of the *Z. coccineum* was chromatographically separated using L.C.–M.S. resulting in generation of characteristic fragment ions that were identified by the system software, and the relative percentages of the separated compounds were determined. However, it could be noticed that isorhamnetin-3-O-glucoside, tribuloside, 7-acetoxy-4-methyl coumarin, luteolin, and zygophyloside-S were the most common compounds in *Z. coccineum* extract as shown in Table 1 and Figure 1. Moreover, in general, the separated compounds were included into nine phytochemical groups, though fourteen compounds belonged to flavonoids that represented 40.89% of the total LC-MS chromatogram contents. The terpenoid compounds (including saponins) were in second place, representing 36.73% of the total contents detected by 12 compounds. Interestingly, eight quinovic acid-based triterpenoid saponins were detected with a relative percentage value of 16.74% of the total plant extract's constituents that were characteristic of this plant species. Furthermore, five phenolic compounds, including coumarins, represent 16.35% of the total LC-MS chromatogram contents. Also, only two compounds, i.e., cinnamaldehyde and syringaldehyde, were separated as aldehydes (1.94%). The lipid percentage was 1.82% of the total contents, and it contained two sterols as well as one fatty acid. Alkaloids (0.55%), alcohols (0.34%), organic acids (0.24), and anthocyanins (0.07%) were also presented as minor contents.

### 3.2. Effect of *Z. coccineum* Extract on Lipoxygenase Enzymatic Activity

**Figure 2:**
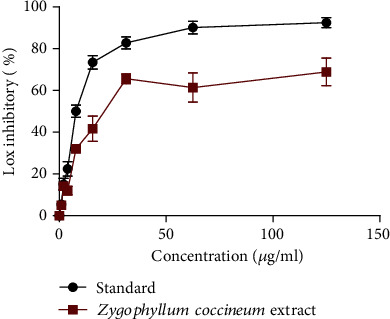
Graphical representation of in vitro anti-inflammatory assay of *Z. coccineum* extract. Results are expressed as a mean ± SEM (*n* = 3), where (IC_50_ = 15.6 ± 2.1 *μ*g/ml for *Z. coccineum* extract while, IC_50_ = 1.4 ± 1.2 *μ*g/ml for standard).

*Z. coccineum* extract showed promising inhibition of lipoxygenase action. The IC_50_ value for 15.6 ± 2.1 *μ*g/ml, whereas for ibuprofen as reference compound, it was estimated as IC_50_ value of 1.4 ± 1.2 *μ*g/ml as shown in Figure 2.

### 3.3. Effect of *Z. coccineum* Extract on Paw Volume and Arthritis Score

**Figure 3:**
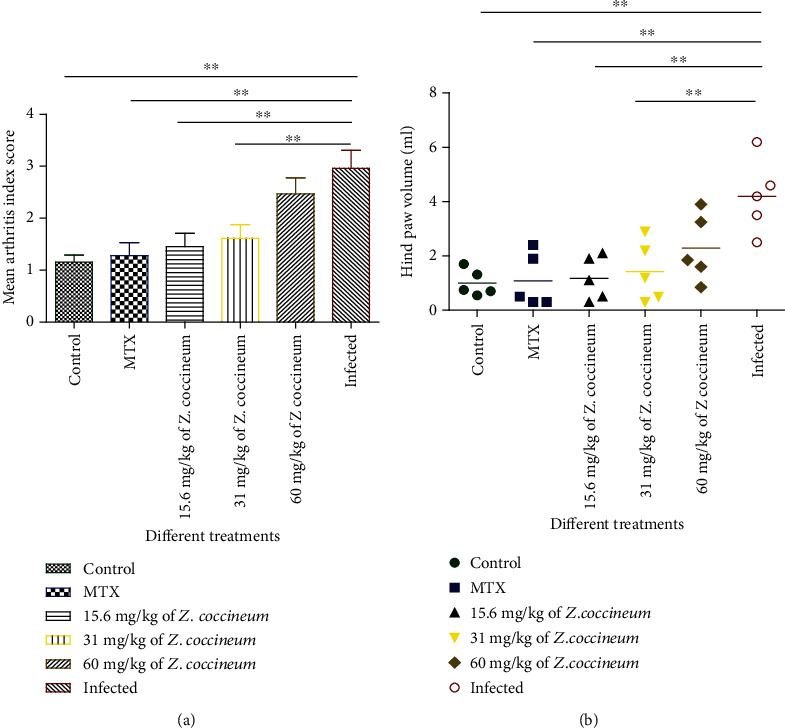
Effect of 15.6, 31, and 60 mg/kg of *Z. coccineum* extract on (a) arthritis index score and (b) paw volume in arthritic rats. The induced elevation in paw volume and arthritis index score was inhibited by 15.6 and 31 *μ*g/ml/kg of *Z. coccineum* extract. The treatment with 60 mg/kg of *Z. coccineum* extract displayed minimal diminution in both paw volume and arthritis index score. Values are represented as mean ± S.E.M. of triplicate values (*n* = 3), where *P* < 0.05.

Paw volume and arthritis score were measured daily after the induction of animals. The paw volume and arthritis score were dramatically increased in the induced rats versus negative control. The paw volume and arthritis score significantly decreased (*P* < 0.05) in the induced animals after receiving 15.6 and 31 mg/kg of *Z. coccineum* extract, similar to obtained results for induced animals and treated with standard drug, while treatment using concentration of 60 mg/kg of *Z. coccineum* extract showed a slight improvement in both tested parameters, indicating that 15 mg/kg of the extract could be significantly improving harmful symptoms of arthritis in the current experimental model, as shown in Figure 3.

### 3.4. Histology and T.E.M. Observations

**Figure 4:**
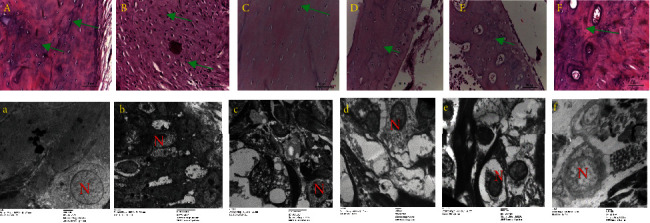
Effects of synovial tissue histopathology of rats with antigen-induced arthritis (magnifications, ×20 and ×8000) using H&E staining and transmission electron microscope. (a, A) Control group with regular chondrocytes (green arrow) with normal nucleus, (b, B) model group with elongated chondrocytes (green arrow), elongated nucleus and many inflammatory cells infiltrated the synovium, (f, F) induced group and receiving MTX as the standard drug group, and cartilaginous tissue could be clearly detected, while (c, C; d, D; e, E) tissues of induced rats showed less inflammation, less lymphocyte accumulation, and regained normal structure of chondrocytes (green arrow) with less cartilage damage decreased upon receiving 15.6, 31, and 60 mg/kg of *Z. coccineum* extract, respectively.

At the end of the experiment (30 days), rats in the negative control group exhibited normal chondrocytes, with a regular sized nucleus and paw cartilage with a flat rough surface as shown in Figure 4(a), A. In the induced rats' extensive inflammatory cells and condensed membrane, deformed nuclei of chondrocytes and clear fat droplets with a score (5) were shown in Figure 4(b), B. The examined results of induced rats upon receiving 15 mg/kg of *Z. coccineum* extract indicated significantly recovering of chondrocyte structure and nucleus size compared to the experimental group as an optimum dose for healing as shown in Figure 4(c), C, while administration of 31 mg/kg of *Z. coccineum* extract for induced rats led to minimal inflammation, as shown through a decreased number of inflammatory cells, thickening, and mild cartilage destruction and slightly altered smaller chondrocytes structure with a score (2-3) as shown in Figure 4(d), D. Furthermore, treating infected animals with 60 mg/kg of *Z. coccineum* extract led to moderate signs of inflammation, reflecting the ineffectiveness of the used dose to overcome the tissue alterations with a score (3) as shown in Figures 4(e) and 4(f). All of these examined results were also compared to the positive control group showing regular tissue structure with mild pathological changes in mitochondria, endoplasmic reticulum, and Golgi bodies, as shown in Figure 4(f), F.

**Figure 5:**
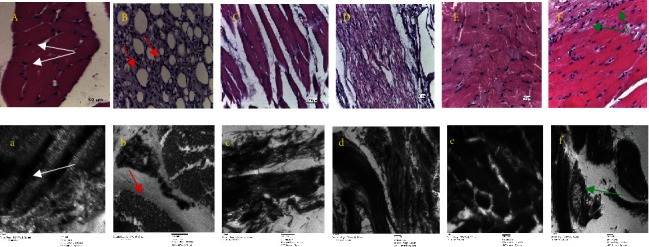
Histology of hind limb muscles, where five groups (a, A) are as follows: control group showing regular muscle structure with elongated nuclei (white arrow). (b, B) Infected with C.F.A. to induce A.I.A. showing injured muscles with severe infiltration and aggregated lymphocytes, macrophages, and plasma cells (red arrow). (c, C; e, E) Induced animals plus 15.6, 31, and 60 mg/kg of *Z. coccineum* extract. (f, F) Induced animals plus treatment with standard drug showing necrosis (green arrow) and slight nuclei aggregation; muscles obtained after animal sacrifice and were stained with H&E and photos captured at a magnification of ×20 and prepared for a transmission electron microscope and images taken at a magnification of ×8000.

To investigate the role of various doses of *Z. coccineum* extract on the morphological alterations in hind limb and muscle, tissues were collected and stained with hematoxylin and eosin and processed for transmission electron microscopy examinations. In the first negative control group, classical muscle structure could be seen with regular fibers and nuclear structure with homogenous distribution, as shown in Figure 5(a), A. There was an increase in inflammation and myofiber degradation in the positive control group along with a decrease in muscle mass and fiber size (Figure 5(b), B). However, by using 15.6 mg/kg of *Z. coccineum* extract for the induced rats, muscle bundles recovered their regular structure as numerous fibers with normal nuclei distribution could be observed as shown in Figure 5(c), C. Furthermore, upon administration of 31 and 60 mg/kg of *Z. coccineum* extract, the examined muscles appeared with a significantly decreased level of cell infiltration and huge irregular fibers with numerous centrally located nuclei as shown in Figures 5(d), D and 5(e), E. In comparison to MTX as a standard drug in induced animals, muscles appeared with slight necrosis and slight aggregations to nuclei, as shown in Figure 5(f), F.

### 3.5. *Z. coccineum* Extract Modulated Osteoclast Activity

**Figure 6:**
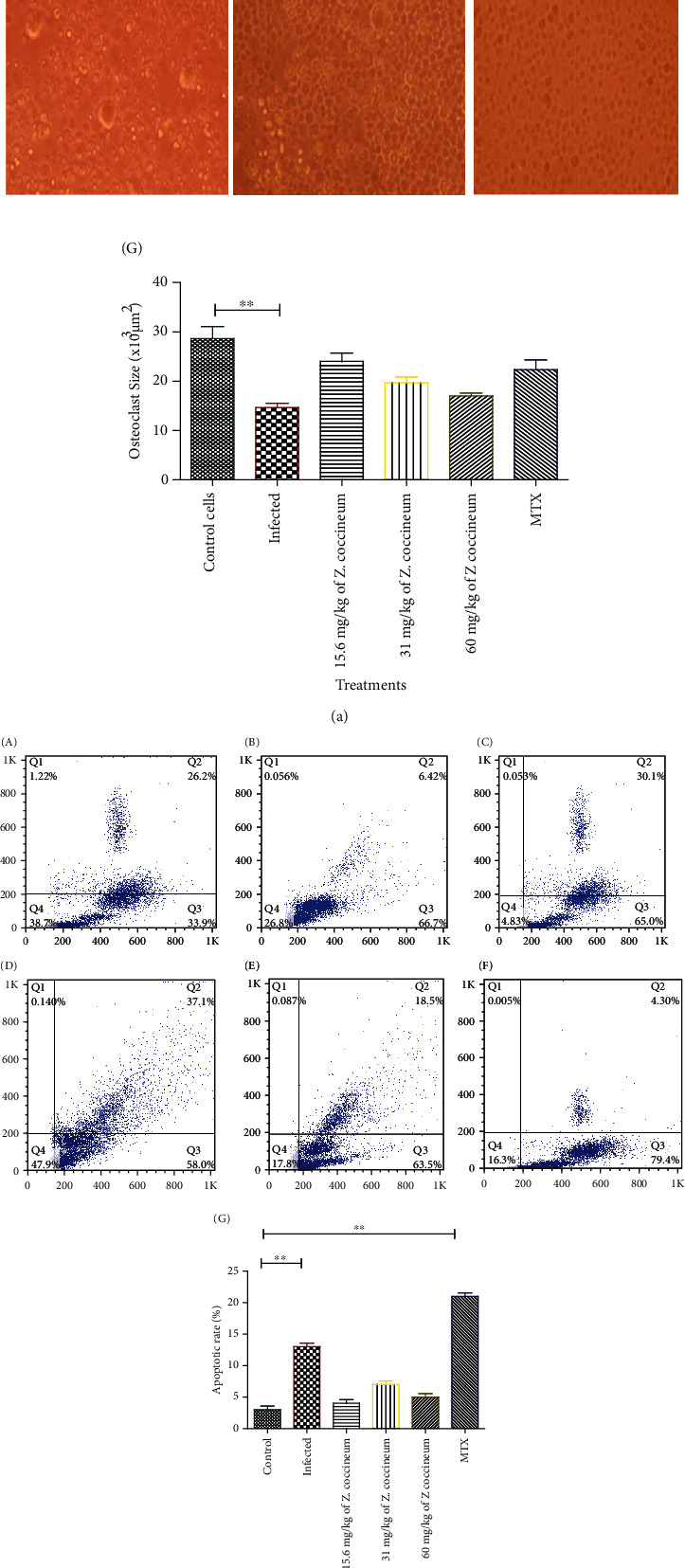
(a) Microscopic examination of osteoclasts in medium enriched with recombinant R.A.N.K.L. Images are representative of cell appearance after the 7 days in culture using phase-contrast microscopy (A) control cells and (B) cells cultured from induced rat. (C)–(E) Cells cultured from induced rat and treated with 15.6, 31, and 60 mg/kg *Z. coccineum* extract, respectively, and (F) cells cultured from induced rat and treated with standard drug where (A) control cells and (B) cells are cultured from induced rat. (C, D, E) Cells cultured from induced rat and treated with 15.6, 31, and 60 mg/kg *Z. coccineum* extract, respectively, and (F) cells cultured from induced rat and treated with standard drug. (G) A bar chart depicts difference in osteoclast size between control group and various treatment; there is a dramatic decrease in osteoclast size in cells cultured cells upon from induced rats (*P* < 0.05), and cells recover their size upon different treatments (15.6, 31, 60 mg/kg *Z. coccineum* extract and MTX). (b) Annexin V-FITC and propidium iodide were used to detect apoptosis in osteoclasts and analyzed by flow cytometer: (A) control cells and (B) cells cultured from induced rat. (C)–(E) Cells cultured from induced rat and treated with 15.6, 31, and 60 mg/kg *Z. coccineum* extract, respectively, and (F) Cells cultured from induced rat and treated with standard drug. (G) A bar chart depicts difference in osteoclast apoptotic rates between control group and various treatment; there is a dramatic increase in osteoclast apoptotic rates in cells cultured cells upon from induced rats and upon using MTX (*P* < 0.05), and cells recover their regular apoptotic rate different treatments (15.6, 31 and 60 mg/kg *Z. coccineum* extract).

To test the effect of the *Z. coccineum* on osteoclasts, isolated osteoclast cells from different groups of animals were cultured for 7 days and examined using an inverted microscope. A significant decrease (*P* < 0.05) could be seen in size of osteoclast cells isolated from induced rats compared to cells isolated cells from the negative control group of animals, while cultured osteoclast cells from animals treated with 15.6 mg/kg of *Z. coccineum* extract resulted in recovering of osteoclast cells its normal structure compared to the negative control group. Furthermore, a slight change could be seen in osteoclast size in isolated cultured cells from animals treated with 31 and 60 mg/kg *Z. coccineum* extract as well as MTX as shown in Figure 6(a). This *ex vivo* result indicated that 15.6 mg/kg of *Z. coccineum* could enhance the propagation of mature osteoclasts in arthritic rats.

To detect apoptotic rate for osteoclasts, Annexin V-FITC kit was used to stain cells. It could be noticed that induction using C.F.A dramatically increased apoptotic rate of osteoclasts (*P* < 0.05). However, using of 15.6, 31, and 60 mg/kg of *Z. coccineum* extract resulted in recovering of osteoclast cells its normal apoptotic rate compared to the negative control group where using of15.6 mg/kg made the optimal recovery. Furthermore, administration of MTX to induced animals increased apoptotic rate of osteoclasts (*P* < 0.05) relative to negative control group as shown in Figure 6(b).

### 3.6. *Z. coccineum* Extract Regulated the Expression of Cytokines

**Figure 7:**
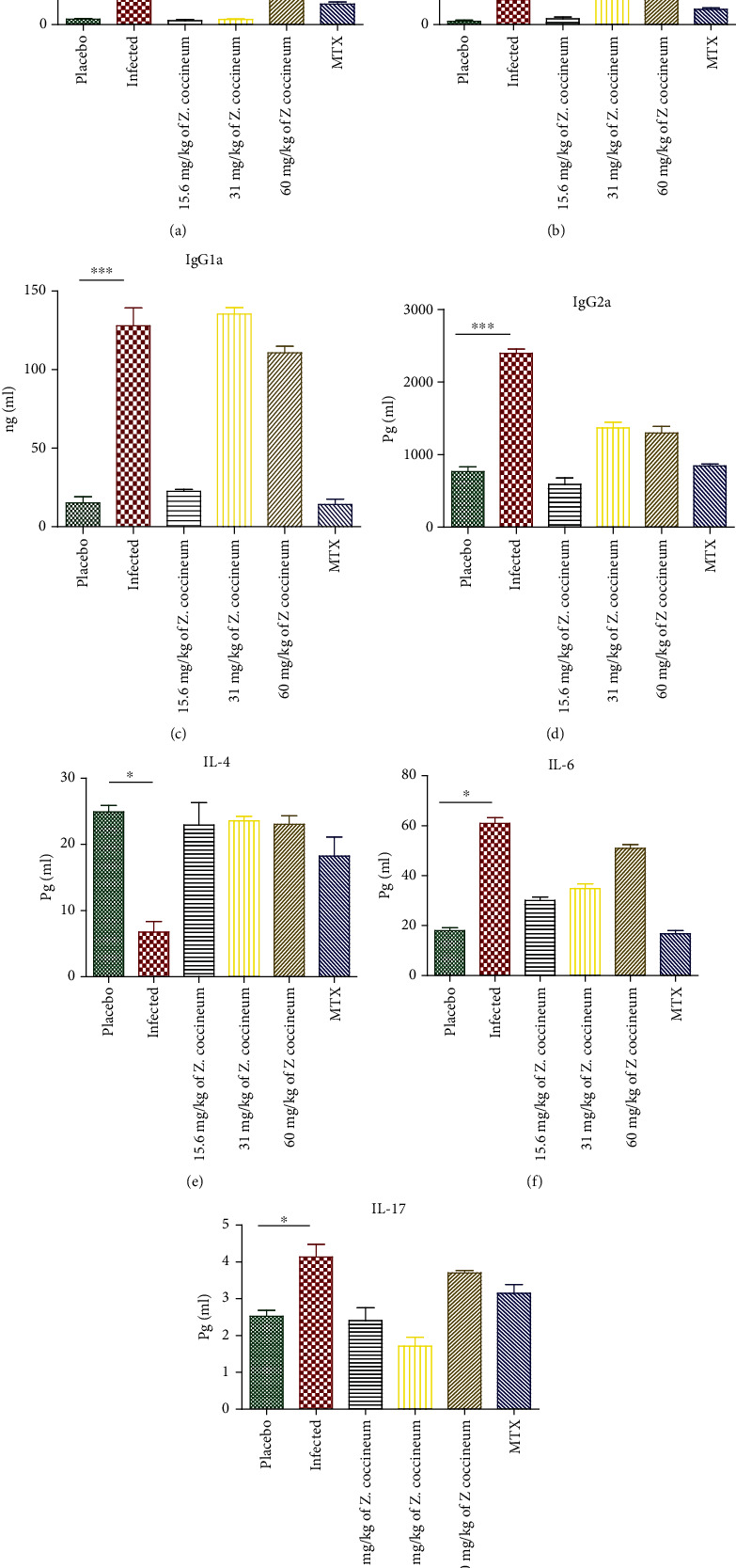
Various concentrations (15.6, 31, and 60 mg/kg) of *Z. coccineum* extract of the levels of cytokines in A.I.A. rats versus control and upon treatment using the standard drug. The level of (a) IFN-*γ*, (b) IL-1*β*, (c) IgG1a, (d) IgG2a, (e) IL-4, (f) IL-6, and (g) IL-17 in rat serum was detected by ELISA. Results are presented as means ± SEM, *n* = 3. ^∗^*P* < 0.05, ^∗∗∗^*P* < 0.01.

ELISA was done to evaluate proinflammatory factors, including IFN-*γ*, IL-1*β*, IL-6, IL-17, and anti-inflammatory factors as IL-4 as well as IgG1a and IgG2a in rat serum. The cytokines levels in serum concentrations of IFN-*γ,* IL-1*β*, IL-6, IL-17, and IgG1a and IgG2a in A.I.A. (antigen-induced arthritis) rats were significantly higher than in control (*P* < 0.05, 0.001). IL-4 declined in serum in A.I.A. rat's (*P* < 0.05). Treatment with 15.6, 31, and 60 mg/kg of *Z. coccineum* extract significantly decreased inflammation by downregulation of IL-1*β*, IL-6, IFN-*γ*, and IL-17 and upregulation of IL-4 in the serum (*P* < 0.05, 0.001). A dose of 15.6 mg/kg *Z. coccineum* extract shows anti-inflammatory action as those of animals treated with MTX as a standard drug as shown in Figure 7. In the current model, regulations of cytokine levels likely lead to the decreased inflammation observed.

### 3.7. Effect of *Z. coccineum* Extract on Various Biochemical Investigations in Rats

**Figure 8:**
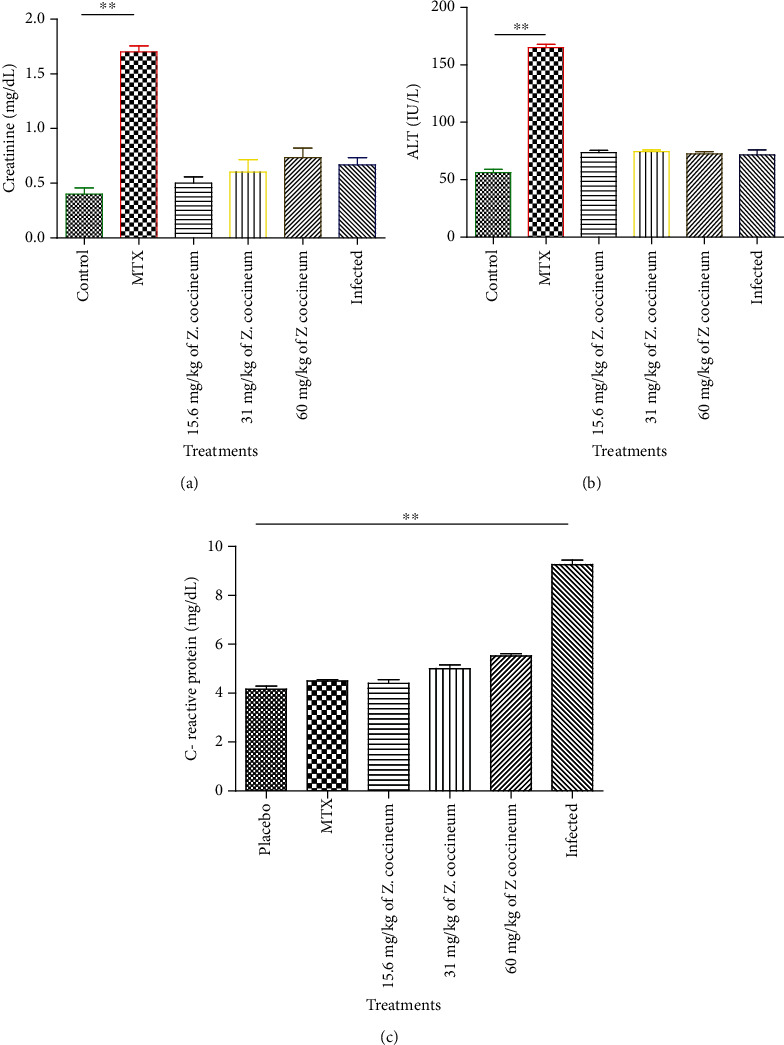
Effect of different concertation (15.6, 31, and 60 mg/kg) of *Z. coccineum* extract on serum liver and kidney functions as well as C-reactive protein in A.I.A. rats versus control and upon treatment using the standard drug. The level of (a) creatinine, (b) A.L.T., (c) C-reactive protein in rat serum was detected by biochemical kits. Results are presented as means ± S.E.M.^∗∗^*P* < 0.05.

The data showed that A.S.T. and creatinine levels were significantly elevated in the group of rats that used MTX as a standard drug for treatment compared to other tested groups (*P* < 0.05), which indicated harmful effects in either kidney or liver functions of this group of tested animals. Furthermore, the induced group of animals using C.F.A. showed a dramatic increase in C-reactive protein level compared to other tested groups (*P* < 0.05). Moreover, upon using 15.6, 31, and 60 mg/kg *Z. coccineum* extract, the C-reactive protein level reached to regular level with slight differences between groups in induced rats, indicating the antiarthritic activity of extract as shown in Figure 8.

## 4. Discussion

Due to the restrictions surrounding human samples from arthritis patients, animal models are a viable alternative to test new therapeutics. [46–48]. Plants contain many compounds that could be used separately or combined in therapeutic applications, including anti-inflammatory functions [49, 50]. In the present study, we test the impact of *Z. coccineum* extract against inflammation symptoms in the antigen-induced arthritis rat model.

In the current study, the separated compounds were included into nine phytochemical groups; flavonoids (40.89%), terpenoid compounds (including saponins 36.73%), phenolic (including coumarins 16.35%), aldehydes (1.94%), lipids (sterols and fatty acid 1.82%, alkaloids (0.55%), alcohols (0.34%), organic acids (0.24), and anthocyanins (0.07%) of the total LC-MS chromatogram contents. However, it could be noticed that isorhamnetin-3-O-glucoside, tribuloside, and 7-acetoxy-4-methyl coumarin were the most common compounds in *Z. coccineum* extract.

Many previous reports recounted the presence of triterpene-saponins, phenolics, and flavonoids in *Z. coccineum* extracts [51, 52]. It has also been reported that *Z. coccineum* possesses potent antioxidant activity and has traditionally been used for hypertension, diabetes, and rheumatoid fever [53].

In this study, the chemotaxonomic marker of the genus *Zygophyllum*, i.e., isorhamnetin-3-O-rutinoside was identified in the *Z. coccineum* ethanolic extract. Tribuloside (also known as kaempferol-3-O-(p-coumaroyl)-glucoside) was detected in the current study as the major flavonoid glycoside constituent as reported by other groups [54–56]. However, the presence of flavonoids and phenolic contents with different structural formulae in *Z. coccineum* extract as obtained by LCMS separation in this study suggested varying roles of the antioxidant activities that was consistent with previously reported by El-Shora et al. [57]. Previously secondary metabolites such as flavonoids derivatives were evaluated in Zygophyllaceae [58]. A recent study on phytochemical screening of Zygophyllaceae family proved its potential as antioxidants and might have a role *in in-vivo* studies as potent therapeutic agents validating their ethnopharmacological usages [59]. Due to their antioxidant capabilities, flavonoids, and phenolic constituents in the plant, suppressing the dangerous oxidative stress and protecting the body from the detrimental free radicals' effects by reducing reactive oxygen species may be essential for biological activities. In accordance with Onguéné et al. [60] who screened the antimicrobial, anti-inflammatory, and antimalarial effects of alkaloids, terpenoids, and coumarin isolated from African plants. Moreover, Matsuda et al. [61] *in vitro* investigated the role of flavonoids stilbene from medicinal plants against Basophilic Leukemia cells. Furthermore, Xu et al. [62] reported the presence of polyphenols and flavonoids from medical plants with anti-inflammatory applications.

Interestingly, eight quinovic acid-based triterpenoid saponins were detected with a relative percentage value of 16.74% of the total plant extract's constituents that were characteristic of this plant species. Moreover, the previous report of Mohammed et al. [32] identified five quinovic acid-based triterpenoid saponins with a 27.12% relative percentage of the total *Z. coccineum* extract's constituents. Also, these types of saponins (quinovic acid-based triterpenoid saponins) were reported from the genus *Zygophyllum* and might be considered a chemical marker for the genus [63].

This study evaluated the effect of using different doses of *Z. coccineum* extract on arthritis score and paw volume to downregulated inflammation signs compared to normal and treatment using the standard drug. Gautam et al. [64] reported the role of bioactive ingredients in butanol fraction of *Punica granatum* in enhancing biophysical arthritic symptoms. Besides, Manan et al. [65] examined the benficial roles of phenolics and flavonoids derived from *Alternanthera bettzickiana* in regulating inflammatory symptoms. Furthermore, Singh et al. [66] explained the oxidative role of phytoconstituents in enhancing arthritic symptoms. In the present report, *Z. coccineum* extract showed promising *in vitro* anti-inflammatory action in accordance with Shahzad et al. [67], who reported antiviral and anti-inflammatory effects of herbs, including *Zygophyllum* growing in Asia and Africa. Besides, Elbadry et al. [68] recorded in vitro antimicrobial and larvicidal activity of Egyptian *Zygophyllum* extract.

The anti-inflammatory action of the Zygophyllaceae family can be attributed to their wide range biological activities [69]. Different species of Zygophyllaceae (*Tribulus longipetalus* and *T. terrestris*) were tested for albumin denaturation assay, and they exhibited significant albumin inhibition and representing significant anti-inflammatory activity [70]. Also, *Tribulus terrestris* extract ameliorated mice macrophages and triggered excessive release of NO and inflammatory cytokines including interleukin-1 beta, interleukin-6, and tumor necrosis factor-alpha [59]. Consequently, we concluded that *Z. coccineum* extract offered an anti-inflammatory effect related to downregulation of NF-*κ*B and mRNA expression inhibition of inflammatory factors including TNF-*α*, IL-1*β*, and IL-6. The inhibition of Akt/M.A.P.K. s is the crucial mechanism suggested the anti-inflammatory activities of natural products [71, 72]. Previously, good anti-inflammatory activity results were documented by Mnafgui et al., [73] for *Z. album* by measuring serum level of C-reactive protein and tumor necrosis factor. Also, the *Z. macropodum* ethanolic extract displayed significant inhibitory activity on increased vascular permeability in mice induced writhing by acetic acid compared to normal control [74].

Previous investigations have also revealed that osteoclasts are cells that have essential roles in bone remodeling [75]. In the current study using of 15.6 mg/kg of *Z. coccineum* extract in induced animals which led to recover osteoclast its regular structure and apoptotic rate, microscopic examinations of joints and muscles revealed that restored chondrocytes and muscles structures restored in induced rats upon using 15.6 mg/kg of *Z. coccineum* extract. Furthermore, Bourebaba et al. [76] *in vitro* investigated the role of *Cladophora glomerata* extract in chondrogenic enhancement. Besides, Saud et al. [77] explained the role of bioactive constituents in controlling gene expression in muscle cells and chondrocytes. Different dose levels of 15.6 mg/kg, 31 mg/kg, and 60 mg/kg *Z. coccineum* extract were tested, and various variations were reported in the current report. In same line with, Amalraj et al. [78] who reported that either low or high doses of curcumin has enhanced signs of inflammtion relative to placebo. However, Antiarthritic activity of 50,100, and 150 mg/kg doses of *Berberis orthobotrys* has been tested in the arthritis animal model where the highest action was seen at 150 mg/kg [79].

Similarly, Lee et al. [69] reported that a compound isolated from a Zygophyllaceae plant *Tribulus terrestris*, known as tribulusamide D, inhibited LPS-induced inflammatory response in RAW264.7 cell model via suppression of NF-*κ*B and P38 signaling cascades. Besides, Ito et al. [80] explained the role of ginger in the differentiation of osteoclasts, while Kim et al. [81] reported the role of *Leonurus sibiricus* in gene expression regulating osteoclast differentiation. In the present study, *Z. coccineum* extract regulated the secretion of pro- and anti-inflammatory cytokines, shifting cytokine profile into protective form. In accordance with Byun et al.; Kulyar et al., [82, 83], who reported the role of medicinal plants in reinforcing immune synapse regulating many diseases. Methotrexate has been used in the treatment of many autoimmune diseases. It has many adverse effects on body organs [84]. Thus, using alternative therapies from natural products has become a significant need in many categories. In the current study, we noticed the beneficial effects of *Z. coccineum* extract in the arthritic model with minimal burden on kidney and liver functions.

## 5. Conclusions

Rheumatoid arthritis (R.A.) is an autoimmune disorder with inflammation in synovium and impacts numerous people all over the world, and there is an emerging trend in using natural products in treatments as safe alternatives. In this study, we have *in vitro* and *in vivo* investigated the anti-inflammatory impact of *Z. coccineum* extract reporting its role in enhancing outer inflammation signs as well as in chondrocytes, osteoclasts, and inflammatory cytokines in a dose-dependent effect with a notable safety effect on the internal body functions as shown in supplement (2) for proposed flow chart of the mechanism. *Z. coccineum* extract could be used as an effective natural source for the treatment of rheumatoid arthritis with high safety profile after more confirmation and verification of results in future studies.

## Figures and Tables

**Table 1:** Identified compounds in the aqueous-ethanolic extract of *Z. coccineum* by L.C.–ESI-TOF–M.S (detected in positive and negative mode of ESI).

No.	R.T. min.	Characteristic mass fragments	Molecular weight (m/z)	Compound name	Chemical formula	%^1^
1	12.69	43, 68, 88	*88.06*	Pyruvic acid	C_3_H_4_O_3_	0.09
2	15.34	105, 133	133.10	Cinnamaldehyde	C_9_H_8_O	0.21
3	16.51	43, 71, 89	134.08	Malic acid	C_4_H_6_O_5_	0.15
4	18.37	93,137	137.02	p-Hydroxybenzoic acid	C_7_H_6_O_3_	0.11
5	24.88	129,146	146.16	Spermidine	C_7_H_19_N_3_	0.16
6	25.71	120, 138, 147, 154	154.16	4-Hydroxy-3-methoxybenzyl alcohol	C_8_H_10_O_3_	0.34
7	26.32	119, 163	163.04	p-Coumaric acid	C_9_H_8_O_3_	0.53
8	26.86	43, 172	172.26	Capric acid	C_10_H_20_O_2_	0.91
9	27.29	135, 150, 161, 179	179.05	Caffeic acid	C_9_H_8_O_4_	1.35
10	30.67	129, 180	180.17	1,7-Dimethylxanthine	C_7_H_8_N_4_O_2_	0.39
11	31.78	123, 140, 168, 183	183.09	Syringaldehyde	C_9_H_10_O_4_	1.73
12	33.19	161, 219	219.15	7-Acetoxy-4-methyl coumarin	C_12_H_10_O_4_	11.07
13	33.24	179, 223	223.13	Sinapic acid	C_11_H_12_O_5_	3.29
14	33.76	153, 219, 263	263.13	Abscisic acid	C_15_H_20_O_4_	2.14
15	34.12	204, 251, 252, 267	267.07	Formononetin	C_16_H_12_O_4_	1.56
16	34.95	119, 121, 153, 225, 271	271.06	Apigenin	C_15_H_10_O_5_	5.19
17	35.77	137, 153, 241, 269, 287	287.05	Luteolin	C_15_H_10_O_6_	10.21
18	36.21	147, 273	273.14	Naringenin	C_15_H_12_O_5_	0.37
19	37.04	153, 286, 301	301.07	Kaempferide	C_16_H_12_O_6_	0.22
20	38.13	153, 165, 229, 247, 285, 303	303.04	Quercetin	C_15_H_10_O_7_	0.37
21	38.41	285, 303	303.09	Taxifolin	C_15_H_12_O_7_	0.43
22	39.72	121, 153, 229, 302, 317	317.06	Isorhamnetin	C_16_H_12_O_7_	0.35
23	40.38	414	414.70	*β*-Sitosterol	C_21_H_24_O_9_	0.42
24	42.65	165, 241 287, 433	433.11	Kaempferol-3-O-*β* –L-rhamnoside	C_21_H_20_O_10_	0.19
25	45.73	43, 456	456.23	Ursolic acid	C_30_H_48_O_3_	0.36
26	51.28	302, 317,479	479.12	Isorhamnetin-3-O-glucoside	C_22_H_22_O_12_	21.14
27	52.17	229, 247, 303, 465	465.14	Hyperoside (quercetin-3-O-galactoside)	C_21_H_20_O_12_	0.38
28	52.86	414, 576	576.85	*β*-Sitosterolglucoside	C_35_H_60_O_6_	0.49
29	54.79	287, 433,579	579.14	Kaempferol3,7-di-O-*β* -L-rhamnoside	C_27_H_30_O_14_	0.09
30	57.18	287, 449, 595	595.16	Cyanidin-3-O-rutinoside	C_27_H_31_O_15_	0.07
31	57.93	153, 229, 303, 465, 611	611.16	Rutin	C_27_H_30_O_16_	0.12
32	59.04	300, 315, 623	623.28	Isorhamnetin-3-O-rutinoside	C_28_H_32_O_16_	0.27
33	61.58	602, 647	647.37	3-O-[*β*-D-Glucopyranosyl] quinovic acid	C_36_H_56_O_10_	0.14
34	61.87	285, 593	593.15	Tribuloside	C_30_H_26_O_13_	17.35
35	64.72	485, 631, 763	763.02	3-O-[*α*-L-Arabinopyranosyl-(1 → 2)-*β*-D-quinovopyranosyl] quinovic acid	C_40_H_62_O_14_	0.43
36	65.83	254, 587, 749, 793	793.44	3-O-[*β*-D-Quinovopyranosyl] quinovic acid-28- *β* -D-glucopyranosyl ester	C_42_H_66_O_14_	0.28
37	67.71	254, 587, 749, 803	803.41	Zygophylloside S (3-O-[*α*-L-arabinopyranosyl-(1 → 2)-*β*-D-glucopyranosyl] quinovic acid)	C_41_H_64_O_14_	6.53
38	69.28	603, 647, 809	809.43	Zygophyloside-K (3-O-[*β*-D-glucopyranosyl] quinovic acid-28-O-*β*-D-glucopyranosyl ester)	C_42_H_66_O_15_	3.48
39	70.23	587, 667, 711, 873	873.40	Zygophyloside-F	C_42_H_66_O_17_	2.32
40	71.65	97, 727, 845,889	889.39	Zygophyloside-G	C_42_H_66_O_18_	1.19
41	72.89	603, 647, 809, 891	891.24	3-O-[*α*-L-Arabinopyranosyl-(1 → 2)-*β*-D-quinovopyranosyl] quinovic acid-28-O-*β*-D-glucopyranosyl ester	C_42_H_66_O_15_	1.17
42	73.27	602, 893	893.13	3-O-[*β*-D-(2-O-Sulphonyl)-quinovopyranosyl] quinovic acid	C_42_H_66_O_14_S	1.34

^1^Relative percentages of compounds in *Z. coccineum* extract were calculated based on the total peak area in the chromatogram.

## Data Availability

All data that support the findings of this study are available from the corresponding authors upon reasonable request.

## Supplementary Materials

Supplementary Materials Supplement (1): various groups of animals and different treatments using Z. coccineum extract in the A.I.A model. Supplement (2): flow diagram illustrating proposed various cells playing some roles in treatment of A.I.A after using Z. coccineum extract.
