# Supplementary material for: Determination of Therapeutic and Safety Effects of Zygophyllum coccineum Extract in Induced Inflammation in Rats
Source: Biomed Res Int. 2022 Jul 18;2022:7513155. doi: 10.1155/2022/7513155 (PMC9314163; doi:10.1155/2022/7513155)
Supplement: Supplementary Materials — Supplement (1): various groups of animals and different treatments using Z. coccineum extract in the A.I.A model. Supplement (2): flow diagram illustrating proposed various cells playing some roles in treatment of A.I.A after using Z. coccineum extract. [file 7513155.f1.zip › Supplement (1) (1).pdf]

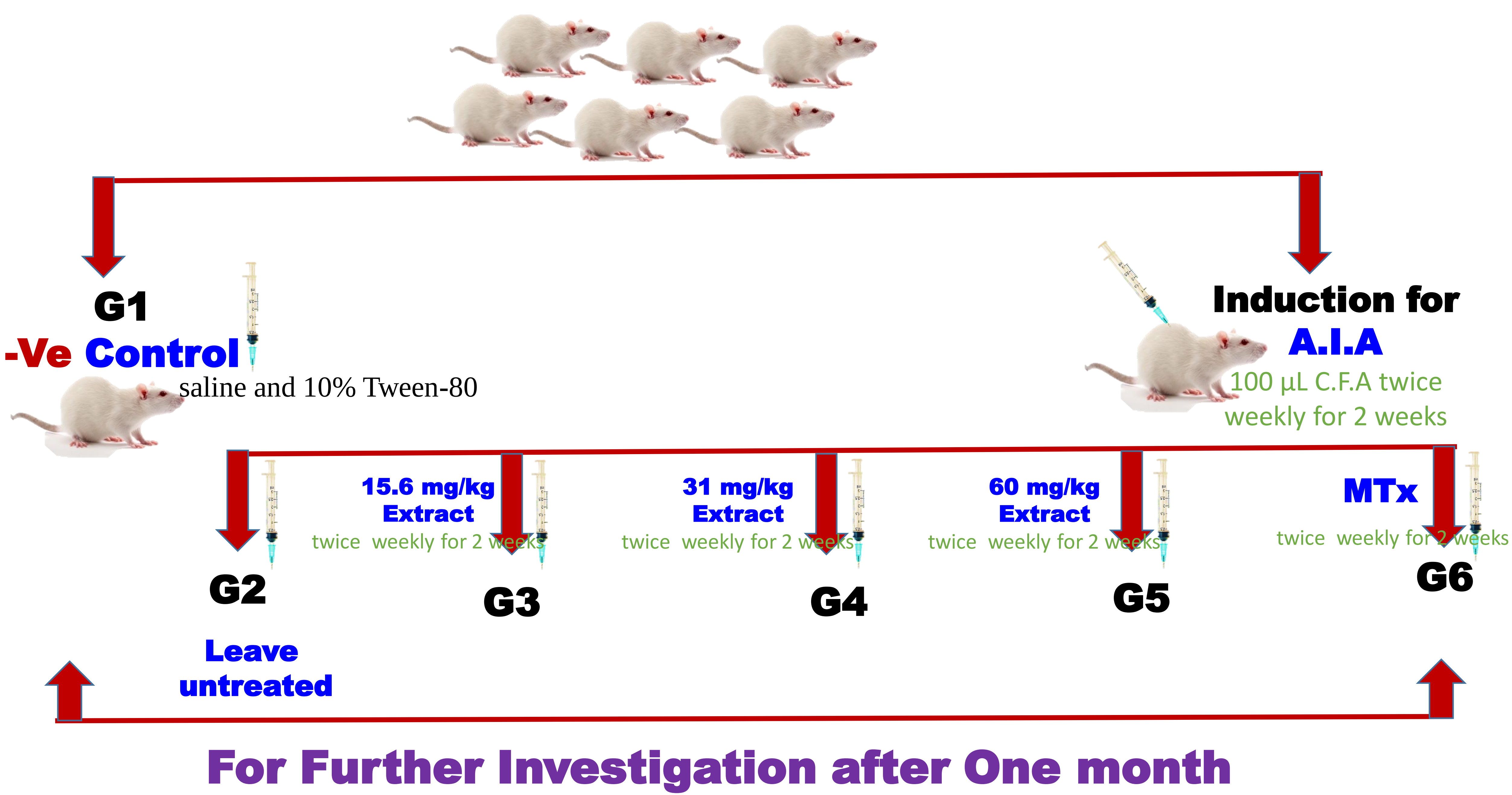
G1
-Ve Control
saline and 10% Tween-80
Induction for
A.I.A
100 µL C.F.A twice
weekly for 2 weeks
15.6 mg/kg
Extract
twice weekly for 2 weeks
31 mg/kg
Extract
twice weekly for 2 weeks
60 mg/kg
Extract
twice weekly for 2 weeks
MTx
twice weekly for 2 weeks
G2
G3
G4
G5
G6
Leave
untreated
For Further Investigation after One month
