# Supplementary figures and images for: Determination of Therapeutic and Safety Effects of Zygophyllum coccineum Extract in Induced Inflammation in Rats
Source: Biomed Res Int. 2022 Jul 18;2022:7513155. doi: 10.1155/2022/7513155 (PMC9314163; doi:10.1155/2022/7513155)

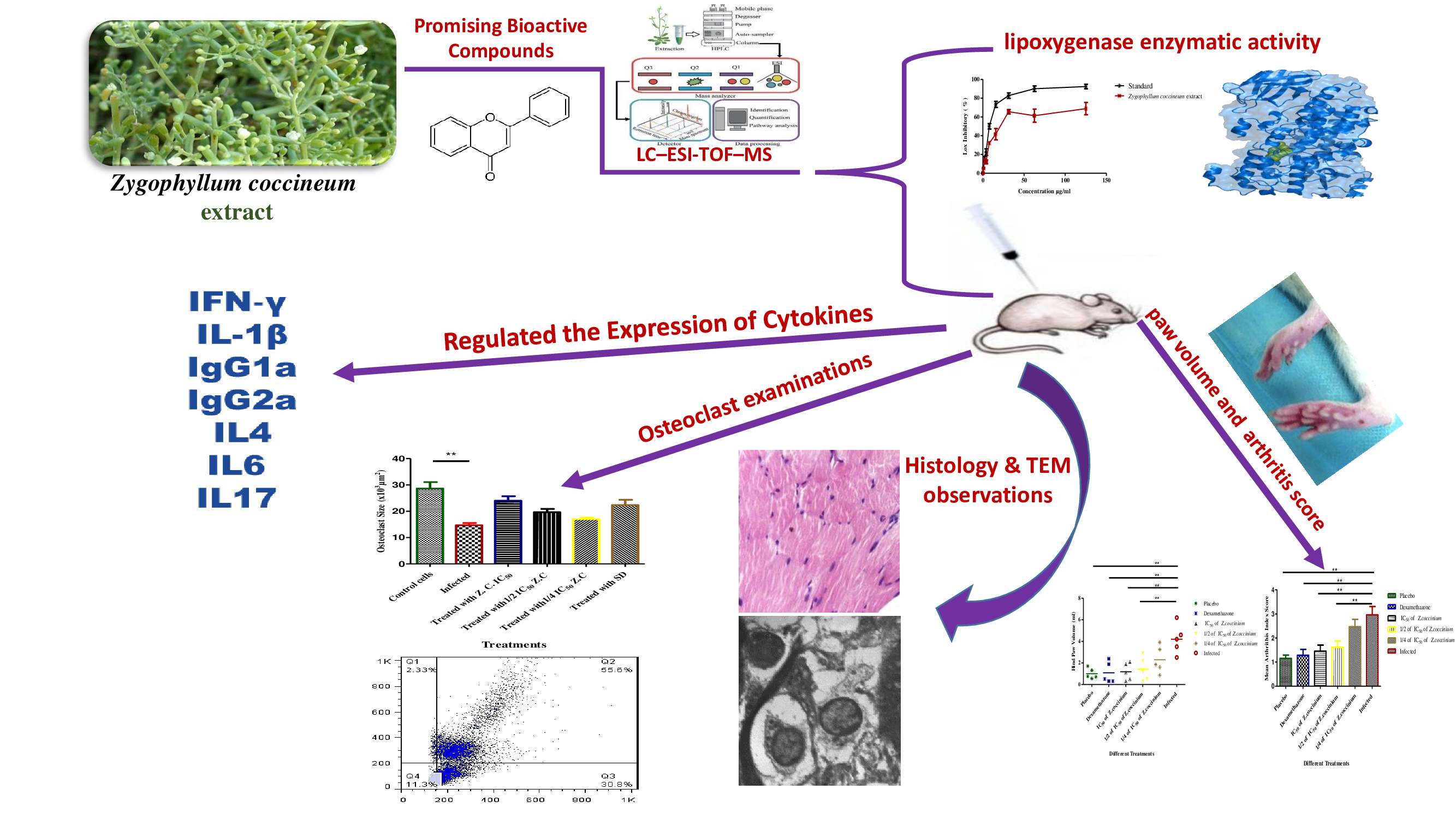

Supplement: Supplementary Materials — Supplement (1): various groups of animals and different treatments using Z. coccineum extract in the A.I.A model. Supplement (2): flow diagram illustrating proposed various cells playing some roles in treatment of A.I.A after using Z. coccineum extract. [file 7513155.f1.zip › Graphical-Abstract (1).jpeg]

# Proposed Mechanism Flow chart

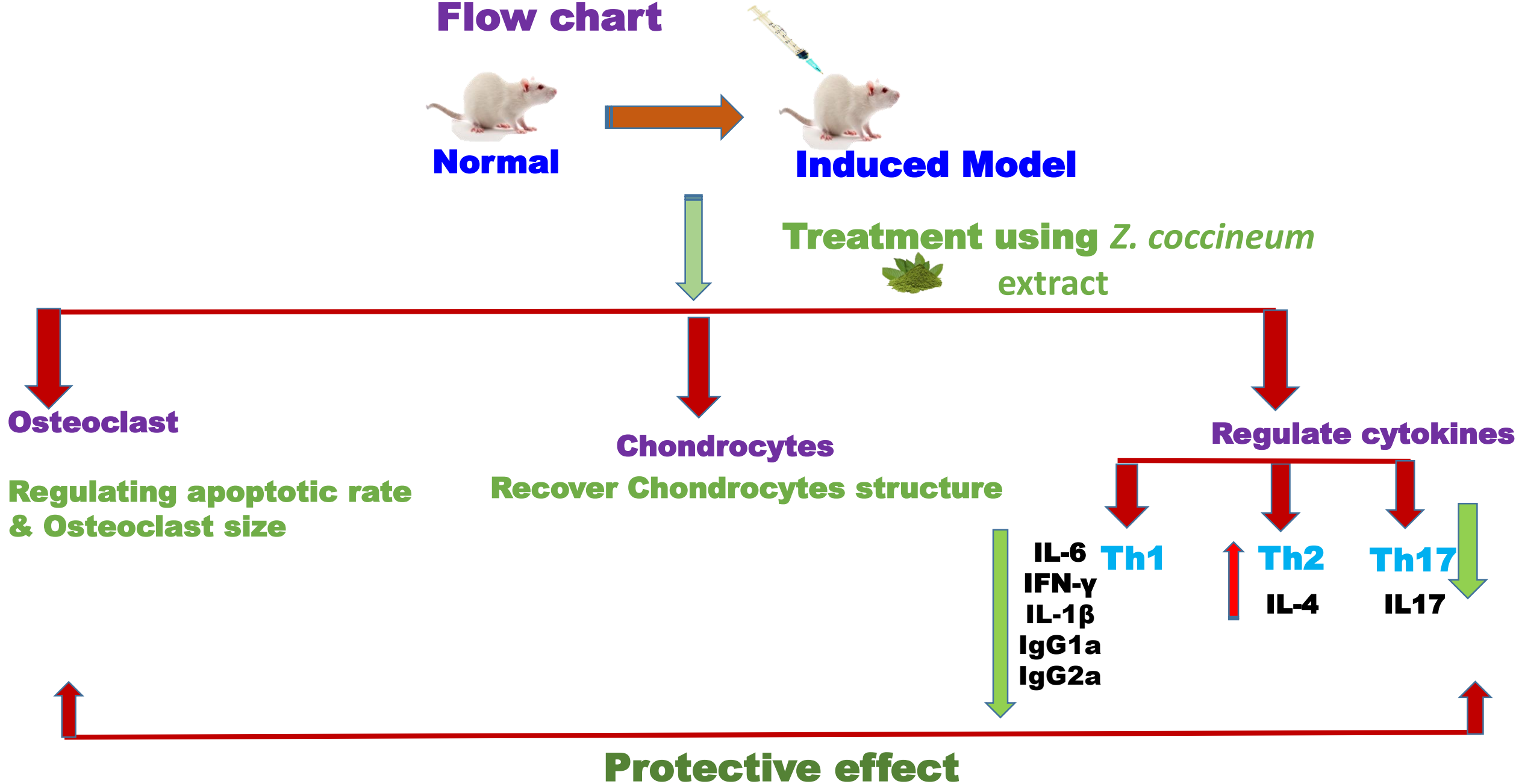

Supplement: Supplementary Materials — Supplement (1): various groups of animals and different treatments using Z. coccineum extract in the A.I.A model. Supplement (2): flow diagram illustrating proposed various cells playing some roles in treatment of A.I.A after using Z. coccineum extract. [file 7513155.f1.zip › Supplement (2) (1).pdf]
